# Rare Variant of a Giant Compound Odontoma of the Maxilla: A Case Report

**DOI:** 10.7759/cureus.63193

**Published:** 2024-06-26

**Authors:** Netal Kela, Gaurav Khemka, Amit Reche, Hitesh Taori, Prasanna R Sonar, Mahek R Batra

**Affiliations:** 1 Public Health Dentistry, Sharad Pawar Dental College, Datta Meghe Institute of Higher Education and Research (Deemed to be University), Wardha, IND; 2 Oral Surgery, Shree Narayana Hospital, Raipur, IND; 3 Public Health Dentistry, Sharad Pawar Dental College and Hospital, Datta Meghe Institute of Higher Education and Research (Deemed to be University), Wardha, IND; 4 Oral and Maxillofacial Surgery, Vayam Hospital, Bhilai, IND; 5 Oral Medicine and Radiology, Sharad Pawar Dental College and Hospital, Datta Meghe Institute of Higher Education and Research (Deemed to be University), Wardha, IND; 6 Dentistry, Sharad Pawar Dental College And Hospital, Datta Meghe Institute of Higher Education and Research (Deemed to be University), Wardha, IND

**Keywords:** imaging, maxillary canine, histopathology, surgical excision, tooth eruption, s: compound odontoma

## Abstract

Benign odontogenic tumors that produce features resembling teeth are known as compound odontomas. This case report describes a unique case of a compound odontoma presenting in the patient's maxillary, the front region. This example highlights how crucial it is to diagnose and treat odontomas as soon as possible in order to avoid possible consequences like tooth displacement or failed eruption. It also emphasizes how important imaging methods are to accurately localizing and planning surgery for odontogenic tumors. The effective handling of this case offers important insights into the multidisciplinary approach needed for odontoma patient diagnosis, treatment, and follow-up.

## Introduction

Odontomas are thought to be developmental abnormalities brought on by the proliferation of mesenchymal and epithelial cells that have undergone full differentiation and give rise to odontoblasts and ameloblasts. In 1866, the word “odontoma” was coined by Paul Broca [1]. A tumor is characterized by the proliferation of all of the abnormal cells. Odontomas are considered to be hamartomatous. Odontomas may be categorized into two types based on the WHO classification, 2005: complex and compound odontomas [2,3]. When the tissues of the teeth are healthy and aligned systematically, they are said to have compound odontomas. On the other hand, complex odontomas are seen in tissues of the oral cavity that are properly formed yet exhibit an amorphous and rather disordered organization. These conditions result in multiple small elements that resemble teeth, called odontoids or denticles, that are differentiated from one another [2,3]. A comprehensive understanding of this odontogenic tumor's diagnosis, therapy, and management can be acquired from the case report on compound odontoma of the maxilla.

## Case presentation

**Figure 1:**
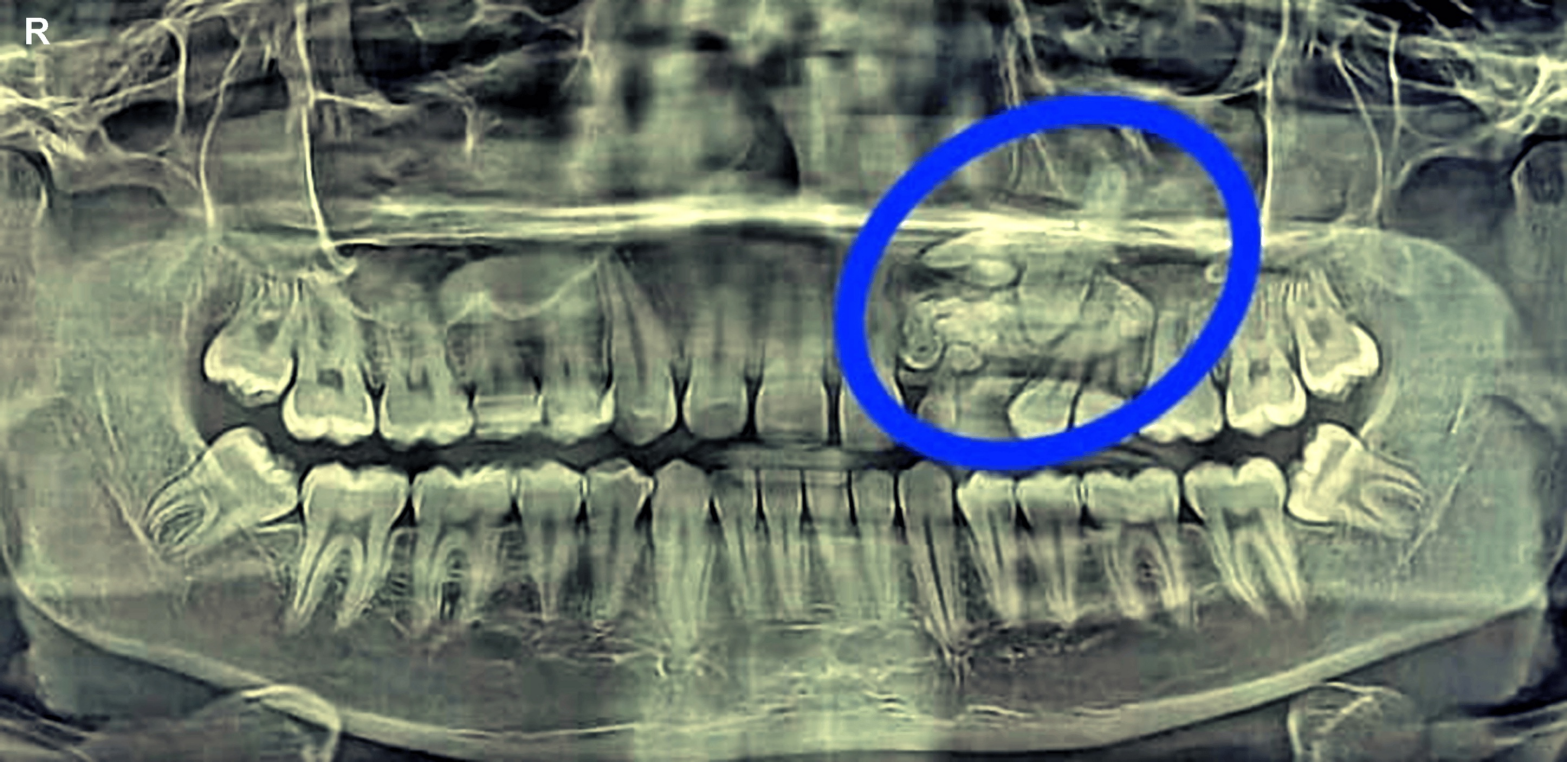
Preoperative OPG of the patient OPG: Orthopantomogram

**Figure 2:**
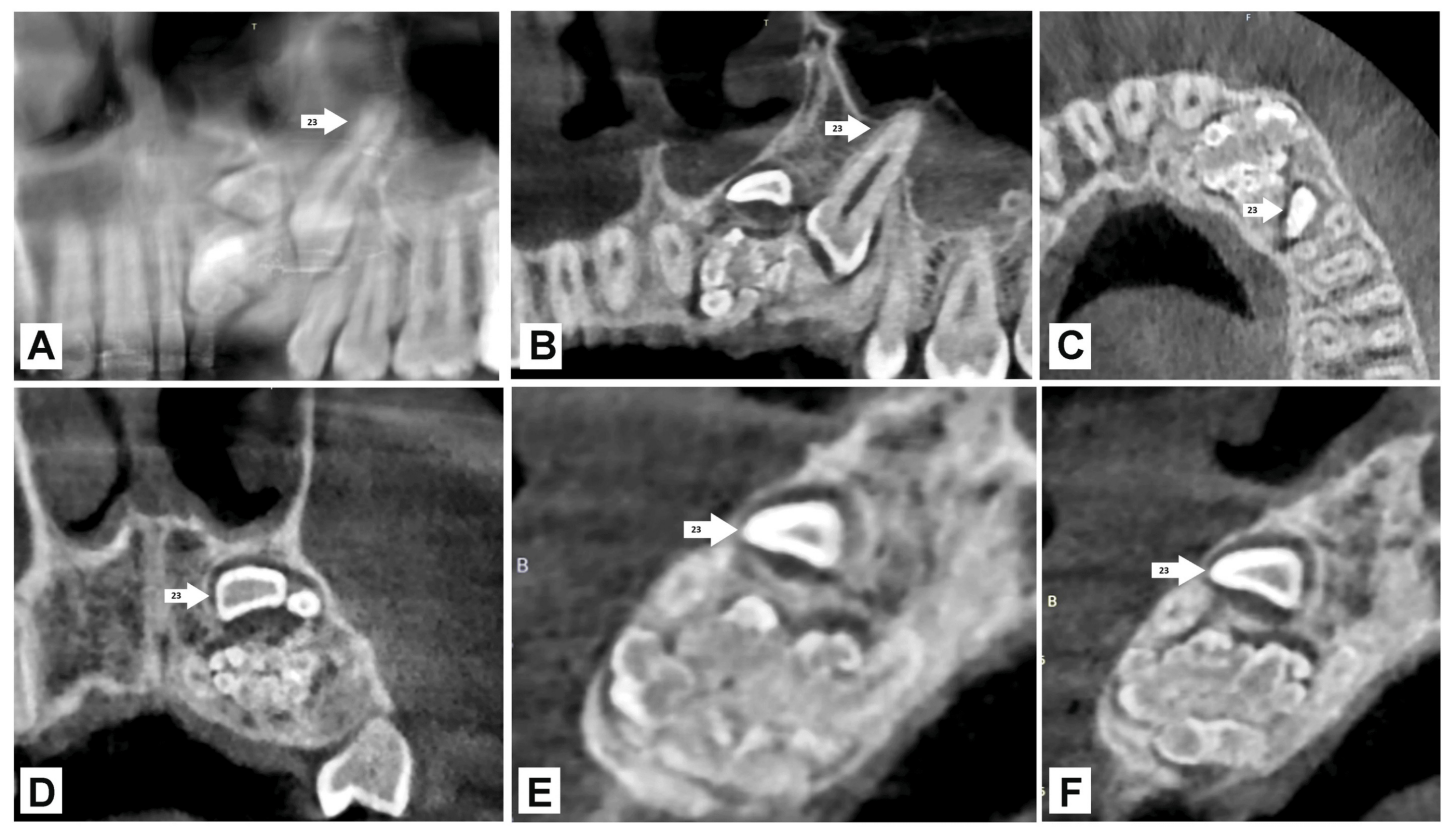
Preoperative CBCT with 22, 23 regions: A) Panoramic image, B) coronal section, C) axial, D) coronal section, E) cross section, and F) cross section Image credit: Netal Kela CBCT: Cone-beam computed tomography

A 17-year-old male patient arrived with a primary complaint of having an unsightly appearance due to delayed permanent teeth eruption in the maxillary left anterior region. Past medical history was not significant. He did not have any prior history of orofacial trauma. Hypodontia or unerupted teeth were not inherited. There was no swelling or tenderness felt upon palpation. Intraoral examination revealed the absence of maxillary left lateral incisor and canine. Examining the hard tissue revealed that all of the permanent teeth in the upper and lower arches were present, except the maxillary left lateral incisor and canine. The provisional clinical diagnosis of compound odontoma was made. After that, the case was assessed by radiographic examination to confirm the diagnosis and pinpoint the exact site and extent of the lesion. The orthopantomogram (OPG) and cone-beam computed tomography (CBCT) showed several radiopaque regions that overlapped like teeth to the impacted maxillary left canine and lateral incisor and a clearly defined periphery with a narrow rim that was radiolucent, as shown in Figure 1 and Figure 2. Complete loss of cortical bone was evident with the 22,23 region with missing 22,23. Based on radiographic and clinical aspects, compound odontoma was made as the diagnosis, with the radiographic findings supported by the clinical findings.

**Figure 3:**
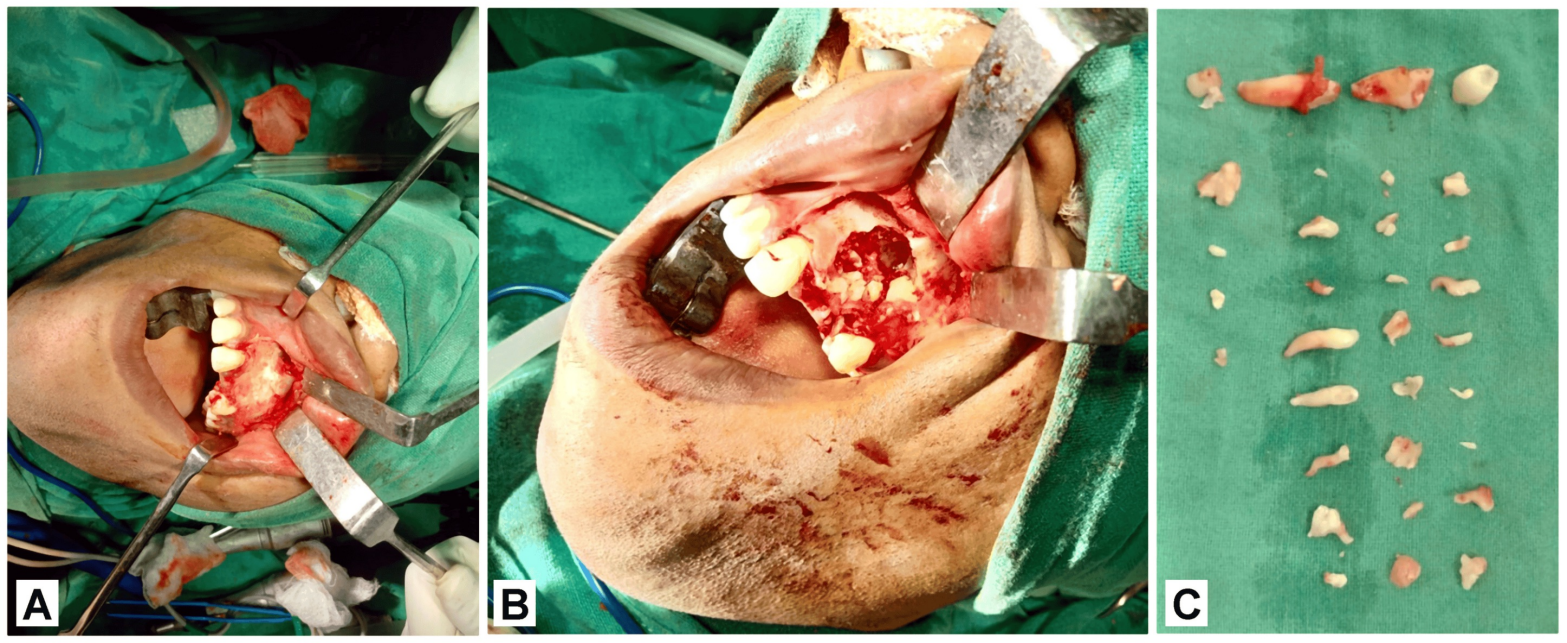
A) Raised mucoperiosteal flap, B) excision of oral tumors, and C) excised odontomas of the patient

A mucoperiosteal flap was raised to create intraoral access while adhering to strict general anesthesia and sterile precautions, as illustrated in Figure 3A. The fibrous capsule along with the calcified structure which was excised into pieces were entirely extirpated, while maintaining constant irrigation with saline solution, all without causing any damage to the surrounding structures, as illustrated in Figure 3B. Along with the odontomas, the affected maxillary left lateral incisor and canine (22,23) were removed. In total, 20 odontomas were removed from that site as shown in Figure 3C. With povidone iodine saline solution, the surgery site was irrigated and curetted. Following the establishment of hemostasis, the flap was placed in its normal position by using 3.0 silk sutures.

**Figure 4:**
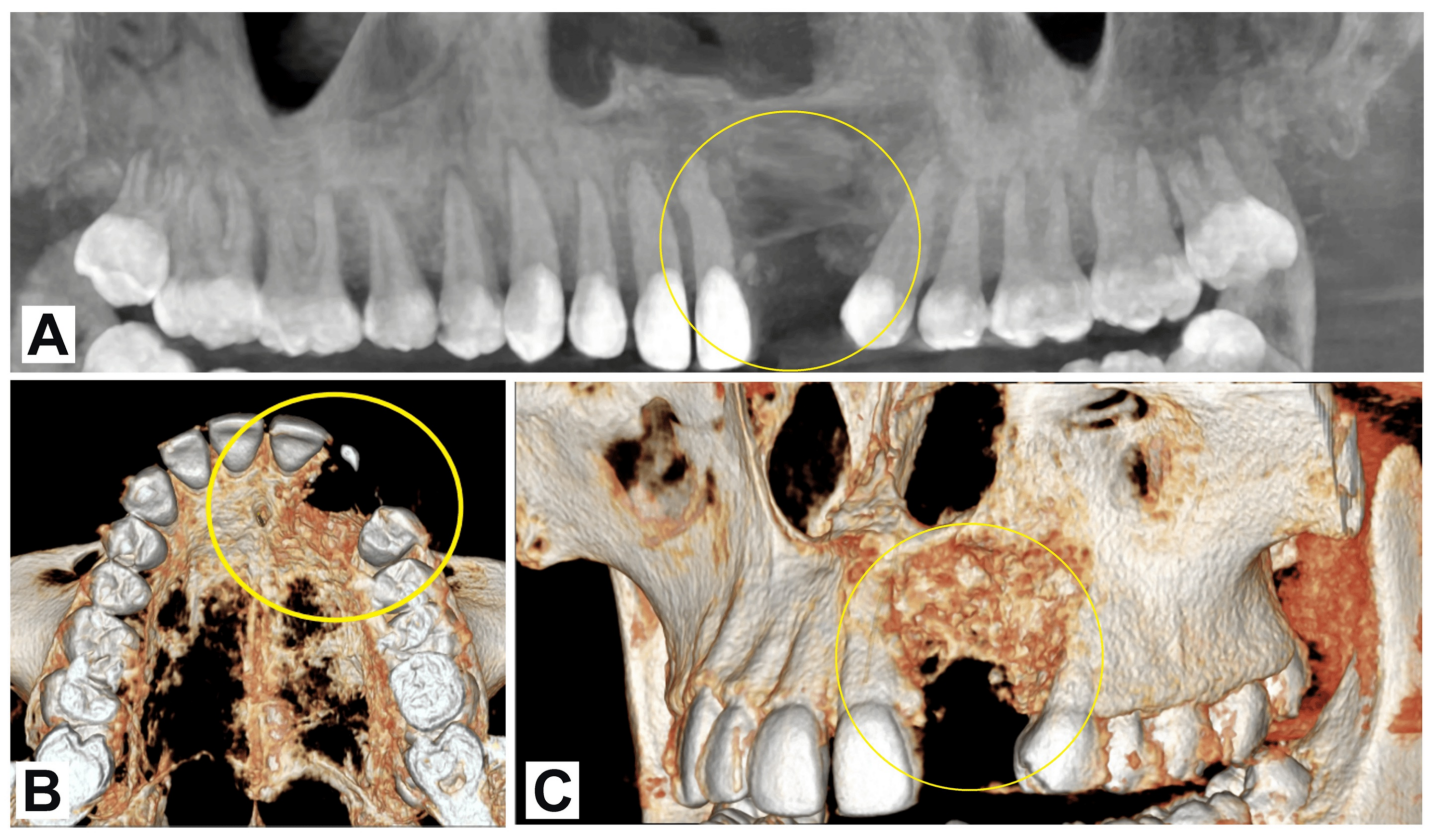
Postoperative follow-up CBCT: A) Panoramic view, B) 3D reconstruction-occlusal view, and C) reconstruction-frontal view CBCT: Cone-beam computed tomography

The patient was advised CBCT for postoperative evaluation to confirm the healing of the affected area. There were no signs of infection clinically and radiographically. Figure 4 shows the postoperative follow-up CBCT of the patient. The patient was monitored regularly, and after a year, there were no complications reported. The patient refused to go through additional dental rehabilitation because of the anxiety he had during the procedure.

## Discussion

Odontomas are asymptomatic odontogenic hamartomatous abnormalities that are somewhat prevalent. Males are more likely than females to have odontomas (59%), and the 2nd decade of their life has a higher frequency rate [4]. Compound odontomas are more prone to the anterior region of the maxilla, mainly the incisor-canine region, whereas the posterior region of both the jaws which is the premolar and molar region is more vulnerable to complex odontomas. Odontomas are typically asymptomatic; they can be unintentionally discovered during a normal radiograph, or when they become sufficiently huge to trigger the jaw to expand and can be seen. A retained deciduous or impacted tooth are clinical indicators that point to an odontoma. The majority of odontomas don't cause any symptoms. They rarely result in tooth displacement, suppuration, edema, discomfort, or bone enlargement. Odontomas have a wide range of dimensions from a few millimeters to centimeters [1-6]. Odontomas are more common in permanent teeth and less common in primary teeth. Three clinical forms of odontomas are currently identified: erupted, peripheral (extraosseous), and central (intraosseous) [1,2]. The erupted odontoma is an intraosseous odontoma that erupts into the oral cavity, while the peripheral odontoma only grows in the soft tissue around the jaw's tooth-bearing region. It is normally seen at 14 years of age, and the 2nd decade of life has a higher frequency rate. Males are more likely than females (41% vs. 59%) to experience this phenomenon. The maxilla (67%) is the most common site affected, and the anterior region (61%) is the most prevalent site. The mandible (33%) is less affected as compared to the maxilla [1,2,5-7].

On radiographs, compound odontoma appears as many radiopaque lesions with different sizes and shapes with toothlike structures called denticles. Complex odontomas are characterized by radiopaque solid mass with fine radiotransparent zones. The lesions are unilocular, and a distinct corticalization line divides them from the healthy bone. Compound odontomas have a high degree of morphodifferentiation, producing a lesion often surrounded by a fibrous capsule and composed of several toothlike features. Odontomas constitute 22% of all odontogenic tumors. The degree of mineralization and development stage determine the radiological results of odontomas. Radiolucency is a characteristic of the early phases due to the lack of calcification. The appearance of the final phase is mostly radiopaque and is encircled by a radiolucent rim that represents the connective tissue, while the intermediate phase is a mix of radiopaque and radiolucent. Odontomas show up radiographically as distinct, radiopaque masses encircled by a radiolucent halo [2,5,6,8].

It is still unclear what causes odontomas. There are numerous pathological conditions associated with odontomas, including odontoblastic hyperactivity, dental lamina remnants, changes in the genetic component of mature ameloblasts that regulate their development into teeth, local trauma or hereditary anomalies, and inflammatory and/or infectious procedures [9]. Histologically, decalcification causes the enamel to break down and hence is not evident on standard hematoxylin and eosin-stained slides, whereas an odontoma reveals pulpal, cementum, and dentin tissue once stained. According to histopathology, the lesion is made up of dentin, pulp tissue, cementum, and normal-appearing enamel or enamel matrix. These components do not show a normal relationship to one another. The odontoma's connective tissue capsule resembles the tooth follicle enclosing a typical tooth. Ghost cells are seen in about 20% of cases. Surgical enucleation and early identification are the best forms of treatment. Delayed primary tooth exfoliation, delayed permanent tooth eruption or impaction, tooth displacement, root resorption, congenitally missing teeth, and follicular space enlargement are among the frequently occurring clinical issues linked to odontomas [2,4,7].

The preferred treatment for odontomas is surgical enucleation because despite their low growth potential, they should be eliminated due to the varied toothlike characteristics that may make them more susceptible to cystic alterations. According to Kaban, odontomas can be surgically excised to remove them without causing damage to neighboring teeth because a bone septum primarily divides them. There is a favorable prognosis with very minimum chances of relapse [4,5]. Despite being benign, compound odontomas need to be carefully evaluated and managed because they may have an impact on the development of teeth and facial aesthetics.

Only a few cases have been reported [10]. About 30 erupting odontomas have been documented to date, according to a study. The maxilla was the site of nearly half of these instances [11]. In a case report published in 2019, Rana and colleagues described the diagnosis of compound odontoma in a 10-year-old kid following the radiographic extraction of his retained right primary mandibular first molar. Following a surgical excision, a compound odontome was discovered by histological analysis. They came to the conclusion that thorough removal and early detection of odontomas provide a better prognosis [12]. A nine-year-old girl presented with a primary complaint of a delay in the exfoliation of her deciduous teeth, according to a 2013 case study by Salgado and Mesquita [13]. A lesion made up of several radiopaque, tiny entities resembling teeth that were obstructing the eruption of the corresponding permanent teeth was visible on a panoramic radiograph. Under general anesthesia, the lesion was accessible by an intraoral technique, and it was surgically removed. A histopathologic investigation verified the diagnosis [13]. Although some publications do not distinguish between the two maxilla, the majority of experts concur that these lesions actually occur more frequently in the upper maxilla [1-4,8]. As is also evident in our case, the odontoma was situated in the upper jaw. Our instance further supports the previously noted propensity of odontomas to develop in the vicinity of the canines and incisors. It's interesting to note that the right side of the jaw experienced higher rates of both forms of odontomas than the left [14]. Radiographic examination to determine the source of a permanent or retained primary tooth frequently leads to discovery [4]. Adjacent teeth that are impacted are frequently linked to odontomas [1-4,6,8]. Iatrou et al. [15] examined 26 cases of odontomas, and 80.7% of them included impaction of permanent teeth. In our instance, there were impacted teeth as well. A retrospective analysis of 45 odontomas revealed that 15 (33.3%) of them had direct contact with the tooth, while 35 (77.8%) were near at least one tooth [16]. Research indicates that the majority of odontomes arise on the right side of the jaw, yet in our instance, the odontome was observed in the upper left quadrant [15]. Our patient was 17 years old, but the average age of incidence was reported to be 20.3 years [17]. Expanded investigation into the molecular mechanisms that give rise to giant compound odontomas may yield valuable insights into focused therapeutic interventions. Additionally, improved cooperation among healthcare specialists may facilitate more efficient diagnostic procedures and improve treatment results for patients presenting with comparable uncommon pathologies.

## Conclusions

Odontomas are uncommon oral eruptions and are typically linked to both retained and impacted teeth. Odontomas are usual hamartomatous lesions that primarily affect people in their second decade of life and are not gender specific. Clinically, they rarely result in facial asymmetry, are frequently asymptomatic, and may occasionally produce discomfort and swelling. Early detection and appropriate treatment of odontomas are critical for avoiding later craniofacial difficulties and other developmental issues. The knowledge that the odontoma was successfully removed surgically in this instance emphasizes the value of early identification and treatment.

## References

[1] Gervasoni C, Tronchet A, Spotti S (2017). Odontomas: review of the literature and case reports. J Biol Regul Homeost Agents.

[2] Mazur M, Di Giorgio G, Ndokaj A (2022). Characteristics, diagnosis and treatment of compound odontoma associated with impacted teeth. Children (Basel).

[3] Barnes L (2006). World Health Organization classification of tumours: pathology and genetics of head and neck tumours. Ear Nose Throat J.

[4] Nelson BL, Thompson LD (2010). Compound odontoma. Head Neck Pathol.

[5] Neville BW, Damm DD, Allen CM, Chi AC (2002). Oral and Maxillofacial Pathology. Oral and Maxillofacial Pathology.

[6] Mallya SM, Lam EW (2000). White and Pharoah's Oral Radiology: Principles and Interpretation. https://iaomfm.com/wp-content/uploads/Books/Oral-Radiology.pdf.

[7] Junquera L, de Vicente JC, Roig P, Olay S, Rodríguez-Recio O (2005). Intraosseous odontoma erupted into the oral cavity: an unusual pathology. Med Oral Patol Oral Cir Bucal.

[8] Waldron AC (2002). Odontogenic cysts and tumors. Oral and Maxillofacial Pathology.

[9] Hitchin AD (1971). The aetiology of the calcified composite odontomes. Br Dent J.

[10] Rumel A, de Freitas A, Birman EG, Tannous LA, Chacon PT, Borkas S (1980). Erupted complex odontoma. Dentomaxillofac Radiol.

[11] Zhuoying C, Fengguo Y (2019). Huge erupted complex odontoma in maxilla. Oral Maxillofac Surg Cases.

[12] Rana V, Srivastava N, Kaushik N, Sharma V, Panthri P, Niranjan MM (2019). Compound odontome: a case report. Int J Clin Pediatr Dent.

[13] Salgado H, Mesquita P (2013). Compound odontoma—case report. Rev Port Estomatol Cir Maxilofac.

[14] Shafer Shafer, Hine Lavy (2009). Shafer’s Textbook of Oral Pathology. 6th ed. New Delhi: Elsevier.

[15] Iatrou I, Vardas E, Theologie-Lygidakis N, Leventis M (2010). A retrospective analysis of the characteristics, treatment and follow-up of 26 odontomas in Greek children. J Oral Sci.

[16] Kämmerer PW, Schneider D, Schiegnitz E, Schneider S, Walter C, Frerich B, Kunkel M (2016). Clinical parameter of odontoma with special emphasis on treatment of impacted teeth-a retrospective multicentre study and literature review. Clin Oral Investig.

[17] Slootweg PJ (1981). An analysis of the interrelationship of the mixed odontogenic tumors: ameloblastic fibroma, ameloblastic fibroodontoma and odontomas. Oral Surg.

